# Evaluation der Krankenhausalarm- und -einsatzplanung anhand einer Übung eines Massenanfalls von Verletzten

**DOI:** 10.1007/s00101-024-01475-5

**Published:** 2024-10-21

**Authors:** Chris Speicher, Thomas Wurmb, Gerhard Schwarzmann, Christian Zech, Hendrik Jansen, Dirk Weismann, Friedrich Anger, Mila Paul, Andreas Münch, Martina Ohr, Patrick Meybohm, Maximilian Kippnich

**Affiliations:** 1 Klinik und Poliklinik für Anästhesiologie, Intensivmedizin, Notfallmedizin und Schmerztherapie, Universitätsklinikum Würzburg, Oberdürrbacherstraße 6, 97080 Würzburg, Deutschland; 2 Stabstelle Medizinisches Struktur‑, Prozess- und Qualitätsmanagement, Universitätsklinikum Würzburg, Würzburg, Deutschland; 3 Klinik und Poliklinik für Unfall‑, Hand, Plastische und Wiederherstellungschirurgie (Chirurgische Klinik II), Universitätsklinikum Würzburg, Würzburg, Deutschland; 4 Medizinische Klinik und Poliklinik I, Universitätsklinikum Würzburg, Würzburg, Deutschland; 5 Klinik und Poliklinik für Allgemein‑, Viszeral‑, Transplantations‑, Gefäß- und Kinderchirurgie (Chirurgische Klinik I), Universitätsklinikum Würzburg, Würzburg, Deutschland

**Keywords:** Krankenhausplanung, Großschadensereignis, Einsatzübung, Patientenmanagement, Ressourcenmanagement, Hospital planning, Mass casualty incidents, Practical exercise, Patient management, Resource management

## Abstract

**Hintergrund:**

Die Krankenhausalarm- und -einsatzplanung (KAEP) stellt für Krankenhäuser ein wichtiges Werkzeug zur Bewältigung eines Massenanfalls von Verletzten (MANV) dar. Krankenhäuser sind vom Gesetzgeber dazu verpflichtet, eine KAEP zu etablieren und zu schulen. MANV-Übungen eigenen sich als Trainingsinstrument für Mitarbeiter und zur Evaluierung der bestehenden Strukturen. Die KAEP des Universitätsklinikums Würzburg (UKW) wurde im Rahmen einer MANV-Übung anhand zuvor definierter Übungsziele evaluiert.

**Methoden:**

Im Rahmen einer groß angelegten MANV-Übung wurden die Abläufe gemäß MANV-Plan des UKW geübt. Dabei wurden als Übungsziele die Überprüfung von Führungsstruktur, Personaleinsatz, Raumordnung, Sichtung, Patientenfluss und Kommunikation festgelegt. Es wurden vorab mehrere Übungsziele definiert. Das Erreichen der Übungsziele wurde anhand eines anonymisierten Fragebogens ausgewertet.

**Ergebnisse:**

Die KAEP des UKW ist grundsätzlich gut geeignet, einen MANV zu bewältigen, wenn adäquat geschult und trainiert wurde. Optimierungsbedarf zeigte sich v. a. bei der Kommunikationsstruktur, den Zuständigkeiten in den Behandlungsbereichen sowie der Kenntnis der Mitarbeiter über die vorhandenen Alarm- und Einsatzpläne und der vorgehaltenen Materialien.

**Diskussion:**

MANV-Übungen sind gut geeignet, die KAEP zu evaluieren. Das Vorhandensein einer klaren Führungs- und Kommunikationsstruktur stellt ein kritisches und erfolgsentscheidendes Element dar. Gute Kenntnisse über die vorgehaltenen Materialien, die Inhalte der KAEP und eine konsequente Anwendung der im MANV-Plan festgelegten Prozesse sind essenziell für einen koordinierten Ablauf des Einsatzgeschehens. Dies kann durch regelmäßige und verpflichtende Schulungen und Trainings erreicht werden.

**Zusatzmaterial online:**

Die Online-Version dieses Artikels (10.1007/s00101-024-01475-5) enthält die beschriebene Patientenversorgungskarte und den Evaluationsbogen. Bitte scannen Sie den QR-Code.

## Abstract (en)

**Background:**

The hospital emergency plan is an important tool for hospitals in the management of a mass casualty incident. It is a legal requirement for hospitals to have such plans in place and to carry out drills. Emergency drills are a useful tool for the training of staff and for the evaluation of existing structures. The emergency plan of the University Hospital Würzburg (UKW) was evaluated during a disaster drill based on predefined drill objectives.

**Methods:**

The procedures according to the emergency plan of the UKW were practiced during a large-scale disaster exercise. The exercise objectives were defined as: testing the management structure, deployment of personnel, physical organization, triage, patient flow and communication. Several exercise objectives were defined in advance. An anonymized questionnaire was used to evaluate the achievement of the exercise objectives.

**Results:**

When properly trained and practiced the UKW emergency plan is generally well-suited to managing a mass casualty event. Improvements need to be made in the communication structure, responsibilities in the treatment areas and staff knowledge of the existing emergency plans and available material.

**Discussion:**

Mass casualty drills are a good tool for the evaluation of hospital emergency plans. A critical and decisive element for success is the existence of a clear management and communication structure. A good knowledge of the available material, the contents of the emergency plan and the consistent application of the procedures defined in this plan are essential for a coordinated course of action. This can be achieved by means of regular and mandatory training sessions.

## Einleitung

Im Rahmen von Großschadenslagen, Katastrophen oder Gefahrenlagen wie Terror und Amok kann es zu einem unerwarteten, zeitgleichen Aufkommen einer hohen Zahl an Verletzten kommen. Ein solcher Massenanfall von Verletzten (MANV) stellt für alle beteiligten Organisationen, sowohl präklinisch als auch klinisch, eine enorme Herausforderung dar [12, 14, 17]. Gerade zu Beginn solcher Lagen sind personelle und materielle Ressourcen knapp. In MANV-Lagen haben sich Sichtungsalgorithmen zur Priorisierung der Behandlung bewährt [1, 8] und gewährleisten eine bedarfsgerechte Therapie der Patienten sowie eine situationsgerechte Nutzung der initial begrenzten Ressourcen. Im Rahmen der Sichtung werden die Patienten anhand ihres Verletzungsmusters und -grades in Sichtungskategorien (SK I–IV bzw. rot, gelb, grün, blau) eingeteilt und gekennzeichnet [9]. Durch die initiale Festlegung der Behandlungspriorität wird die optimale Verteilung des anfallenden Patientenstroms auf die vorhandenen Ressourcen sichergestellt. Bei Eintreffen in der Klinik sollen alle Patienten einer Eingangssichtung unterzogen und anschließend gemäß ihrer Sichtungskategorie den entsprechenden Behandlungsbereichen (rot, gelb, grün) zugeführt werden. Die Sichtung sollte durch ein sichtungserfahrenes und – trainiertes Team, bestehend aus Arzt, Pflegekraft und Dokumentationsassistenz, erfolgen [1, 9, 13, 14].

Um sich auf Schadenslagen und deren Bewältigung vorzubereiten, sind Krankenhäuser dazu verpflichtet, eine Krankenhausalarm- und -einsatzplanung (KAEP) zu entwickeln und vorzuhalten. Die KAEP beinhaltet Verfahrensanweisungen für zu erwartende Schadenslagen (z. B. MANV) und regelt u. a. folgende Elemente: Alarmierung und Funktion der Krankenhauseinsatzleitung (KEL), Raumordnung, klinische Sichtung, Kommunikation, Logistik und Ressourcenmanagement [1, 2, 11, 14, 15, 17, 18, 20].

Die in der KAEP festgehaltenen Prozesse müssen etabliert, geschult und trainiert werden. Übungen von Schadenslagen dienen dabei nicht nur als Trainingselement für Mitarbeiter, sondern gelten auch als wichtiges Evaluationsinstrument bestehender Strukturen und Krisenpläne. Die im Rahmen einer MANV-Übung gewonnenen Erkenntnisse können verwendet werden, um bestimmte Elemente der KAEP zu optimieren [2, 4, 5, 17]. In Würzburg fand im September 2023 eine große präklinische MANV-Übung, an der sich das Universitätsklinikum Würzburg als aufnehmendes Krankenhaus beteiligte, statt.

## Fragestellung und Übungsziele

Ziel dieser Arbeit war es, die KAEP im Rahmen einer MANV-Übung zu evaluieren, vorhandene Schwachstellen zu identifizieren und die KAEP diesbezüglich zu optimieren.

Getestet wurden die Abläufe gemäß MANV-Plan des Universitätsklinikum Würzburg (UKW) mit Schwerpunkt auf den Aspekten Alarmierung, Führungsstrukturen (KEL und operative Einsatzleitung [opEL]), Personaleinsatz, Raumordnung, Sichtung, Patientenverteilung auf die Behandlungsbereiche und Kommunikation. Hierbei war die zu überprüfende Hypothese, dass der MANV-Plan des UKW, unter der Voraussetzung bekannt und geschult zu sein, geeignet ist, einen Massenanfall von Patienten zu strukturieren und erfolgreich zu bewältigen.

Um die Hypothese bestmöglich zu evaluieren, wurden folgende 5 übergeordnete Übungsziele definiert:

1. *opEL, Führung und Kommunikation*: Die operative Krankenhauseinsatzleitung (opEL) führt den operativen Einsatz strukturiert und kommuniziert engmaschig mit den Abschnittsleitern und anderen Bereichen.
2. *MANV-Plan, inklusive Auftragsblätter*: Alle Beteiligten wenden den MANV-Plan bestimmungsgemäß an und sind in der Lage, die Auftragsblätter korrekt einzusetzen.
3. *Raumordnung und Patientenfluss*: Die Raumordnung ist geeignet, um einen kontinuierlichen Patientenfluss zu gewährleisten.
4. *Sichtung und Sichtungsalgorithmus*: Das Sichtungsteam wendet den innerklinischen Sichtungsalgorithmus schnell, sicher und effektiv an.
5. *Ausstattung und Material*: Alle Beteiligten sind mit der Ausstattung (Material, Führungsmittel, usw.) vertraut und verwenden diese zielgerichtet.

## Methodik

### Struktur und Inhalte der KAEP

Die Krankenhausalarm- und -einsatzplanung des Universitätsklinikums Würzburg ist im Intranet des Klinikums einsehbar und enthält u. a. eine Dienstanweisung zum Vorgehen beim Massenanfall von Verletzen, nachfolgend als MANV-Plan bezeichnet. Dieser beinhaltet folgende Elemente:

- Alarmauslösung,
- Bildung, Zusammensetzung und Aufgaben der operativen Einsatzleitung (opEL),
- personelle Ressourcen und Alarmierung von dienstfreiem Personal,
- Versorgungsstrategie und Raumordnung,
- Sichtung: Sichtungsplatz, Sichtungsteam, Algorithmus, Dokumentation,
- Behandlungsbereiche und Zusammensetzung der Behandlungsteams,
- Kommunikation,
- angeschlossene Funktionsbereiche und Abteilungen wie Zentrallabor, Apotheke, Transfusionsmedizin usw.

Ergänzend zum MANV-Plan werden Auftragsblätter (AFB) vorgehalten, welche, als Checkliste konzipiert, eine Orientierungshilfe für die anfallenden Prozesse darstellen und die Aufgaben der verschiedenen Funktionsbereiche zusammenfassen. Diese werden zusammen mit dem MANV-Plan auch in Druckversion in der Zentralen Notaufnahme (ZNA) vorgehalten, sodass im Ernstfall schnell auf diese zurückgegriffen werden kann, was es auch dem unerfahrenen Anwender ermöglicht, seine Aufgaben unkompliziert nachzuschlagen und anschließend umzusetzen.

### Vorbereitung

Um den Regelbetrieb am Uniklinikum möglichst wenig einzuschränken, wurde die Entscheidung getroffen, nicht auf die reguläre Dienstmannschaft zurückzugreifen, sondern zusätzliches Personal für die Übung einzusetzen. Im Vorfeld der Übung fanden eine Besprechung des MANV-Plans und eine Begehung der Räumlichkeiten sowie der vorgehaltenen Ressourcen und MANV-Wägen statt. Da als Übungsziel die Überprüfung des MANV-Plans und nicht der Status der Durchdringung und der Trainingsstand der Mitarbeitenden gewählt worden war, sollte durch diese Maßnahme gewährleistet werden, dass die Übenden mit dem Einsatzplan des UKW vertraut sind. Es wurden Patientenversorgungskarten (Zusatzmaterial online: ESM 1) entwickelt, um simuliert durchzuführende Maßnahmen an den Mimen zu dokumentieren. Zur Evaluation der Übungsziele wurde ein Fragebogen (Zusatzmaterial online: ESM 2) entworfen. Zusätzlich wurde die Entscheidung getroffen, eine Videodokumentation der Übung anzufertigen und Beobachter für bestimmte Schlüsselfunktionen einzusetzen. Letztere nahmen ebenfalls an der Evaluation teil.

### Auswertung

Die Evaluation der oben genannten Übungsziele erfolgte anhand eines anonymisierten Fragebogens (Zusatzmaterial online: ESM 2), welcher am Ende der MANV-Übung von allen Teilnehmern ausgefüllt wurde. Es wurde die Funktionszugehörigkeit (Arzt, Pflegedienst, Sonstiges; Führungskraft oder operatives Team) und die Diensterfahrung in Jahren erfasst. Das Erreichen der Übungsziele wurde anhand einer Likert-Skala (0: „gar nicht“ bis 10: „vollständig“) beurteilt. Zusätzlich wurde anhand offener Fragen nach förderlichen und hinderlichen Faktoren gefragt und Optimierungsvorschläge erfasst. Die offenen Fragen wurden ausgewertet und wiederkehrende Elemente nach ihrer Wichtung zurückbehalten.

### Übungsablauf

Im September 2023 fand im Würzburger Mainhafen eine groß angelegte, vom Amt für Zivil- und Brandschutz organisierte MANV-Übung mit Schauspielpatienten (Mimen) statt, an der sowohl die Feuerwehr, die Polizei, die Würzburger Rettungsdienste, das Universitätsklinikum Würzburg (UKW) und das Klinikum Würzburg Mitte teilnahmen. Die Übung fand an einem Sonntagvormittag im September statt. Die ZNA des Uniklinikums war für die Dauer der Übung nur für Patienten, die das spezifische Behandlungsspektrum des UKW benötigt hätten, aufnahmebereit. Die Versorgung der übrigen Notfälle wurde im Übungszeitraum durch das Klinikum Würzburg Mitte gesichert. Der reguläre Dienstbetrieb am UKW wurde nicht eingeschränkt. Der zeitliche Ablauf der Übung war wie nachfolgend aufgelistet: 8:15 Uhr Briefing; 10:00 Uhr Beginn der MANV-Übung im Würzburger Hafen; 10:30 Uhr Alarmierung der opEL; 10:40 Uhr Lagebesprechung des Dienstpersonals in der ZNA; 11:00 Uhr Eintreffen des ersten Patienten; 12:15 Uhr Übungsende und anschließendes Debriefing.

**Abb. 1:**
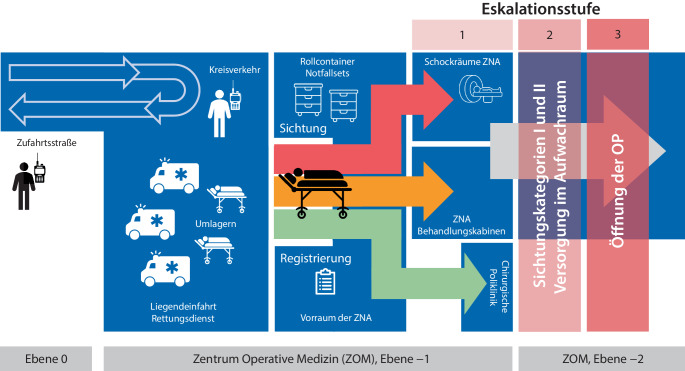
Raumordnung und Patientenfluss gemäß Eskalationsstufen 1–3 des MANV-Plans am Universitätsklinikum Würzburg. Die Etablierung eines geordneten Kreisverkehrs garantiert den Patientenfluss durch Verhindern einer blockierten Zu- und Abfahrt zur ZNA. Gemäß Eskalationsstufe 1 des MANV-Plans findet die Behandlung der Patienten auf Ebene −1 des „Zentrum operative Medizin (ZOM)“, in den Räumlichkeiten der ZNA und chirurgischen Poliklinik (der ZNA gegenüberliegend) statt. Bei Überschreiten der hier vorgehaltenen Behandlungskapazitäten, ist eine sukzessive Erhöhung um 2 Eskalationsstufen laut MANV-PLAN möglich. Bei Stufe 2 wird die Patientenversorgung (nicht jedoch die Sichtung) auf Ebene −2 im Aufwachraum sowie bei Stufe 3 im OP-Trakt (in Einleitungs‑, Ausleitungsräumen, OP) statt. Die Entscheidung über eine Erhöhung der Eskalationsstufe obliegt der opEL bzw. KEL. Im Rahmen der hier vorgestellten Übung wurde lediglich die Eskalationsstufe 1 simuliert

**Abb. 2:**
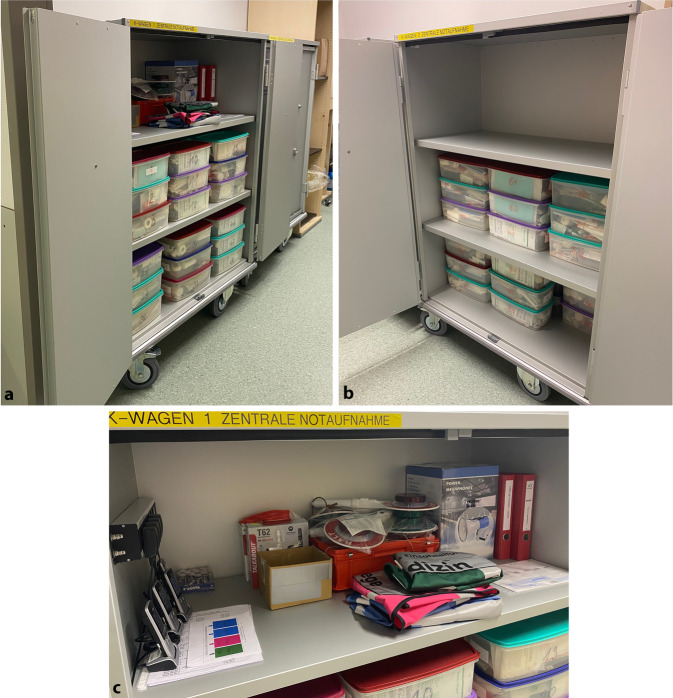
MANV-Wagen (**a**, **b**) und Inhalt (**c**)

Nach Ankündigung eines MANV durch die Integrierte Leitstelle Würzburg wurde die operative Einsatzleitung (opEL), bestehend aus den jeweils diensthabenden Oberärzten der Anästhesiologie, der Allgemeinchirurgie, der Unfallchirurgie sowie der Radiologie, einer ZNA-Pflegekraft und jeweils einem Vertreter der Logistik und der Technik, alarmiert, welche vor Ort in der ZNA die Sichtung und Patientenversorgung koordiniert. Die Leitung der opEL oblag dem Oberarzt der Anästhesiologie. Zeitgleich erfolgte die Alarmierung des Dienstpersonals der im MANV-Plan definierten Fachabteilungen sowie der Krankenhauseinsatzleitung (KEL), der anschließend die Gesamteinsatzleitung obliegt. Sammelpunkt für alle Mitarbeiter war der Wartebereich der ZNA. Nach kurzem Briefing durch die opEL wurden die Behandlungsteams auf die jeweiligen Behandlungsbereiche aufgeteilt. Zudem wurden die Behandlungsbereiche materiell aufgestockt und der MANV-Wagen zum Sichtungsort verbracht. Die Raumordnung ist in Abb. 1 und die Ausstattung des MANV-Wagens in Abb. 2 dargestellt.

Die Einlieferung der Patienten durch den Rettungsdienst erfolgte über die Liegendeinfahrt der ZNA. Da die Patientenzufahrt zur ZNA baulich bedingt nicht für ein erhöhtes Fahrzeugaufkommen im MANV-Fall ausgelegt ist, wird durch Vertreter der Logistikabteilung ein geordneter Kreisverkehr (Abb. 1) etabliert und dadurch sichergestellt, dass die maximale Zahl an Einsatzfahrzeugen in der Entladezone nicht überschritten und somit ein Festfahren sowie ein Erliegen des Patientenflusses verhindert werden. Die Versorgung der Patienten erfolgte gemäß Eskalationsstufe 1 des MANV-Plans in der ZNA, in hierfür räumlich voneinander abgegrenzten Behandlungsbereichen (Abb. 1). Als Sichtungsort wurde planmäßig der Vorraum der ZNA verwendet. Die Eingangssichtung erfolgte, nach Umlagerung des Patienten auf eine Kliniktrage, durch ein erfahrenes Sichtungsteam, bestehend aus einem Facharzt für Anästhesiologie, einer Fachärztin für Unfallchirurgie und einer ZNA-Pflegekraft. Die Sichtungsergebnisse wurden auf dem vorgesehenen Registrierungsbogen und, falls vorhanden, auf der Sichtungskarte notiert. Zur Sichtung wurde der klinikinterne, an den Berliner Sichtungsalgorithmus [13] angelehnte, klinikspezifisch modifizierte Sichtungsalgorithmus verwendet.

Im Klinikinformationssystem existieren 200 für eine MANV-Lage reservierte Fallnummern, welche nach Aktivierung den Patienten bei Registrierung zugeteilt werden können und im Verlauf eine eindeutige Identifizierung der Patienten zulassen. Passend zu diesen Fallnummern gibt es vorgefertigte Patientenarmbänder und Barcode-Aufkleber, welche folgender Systematik folgen: Name: MANV 001 (fortlaufend bis MANV 200), Vorname: Fallnummer. Im Rahmen der Patientenregistrierung wird die entsprechende MANV-Nummer auf einem hierfür vorgesehenen Registrierungsbogen vermerkt und, falls vorhandenen, die Nummer der rettungsdienstlichen Patientenanhängekarte ergänzt. Diese Verknüpfung stellt den entscheidenden Schritt für die spätere Suche, Zuordnung und Nachverfolgung der Patienten aus rettungsdienstlicher Registrierung dar. Patientenarmband mit Fallzuordnung sowie Anhängekarte verbleiben beim Patienten. Sämtliche Dokumentationen, Anforderungen sowie Untersuchungen laufen anschließend über die zugeteilten MANV-Nummern.

**Abb. 3:**
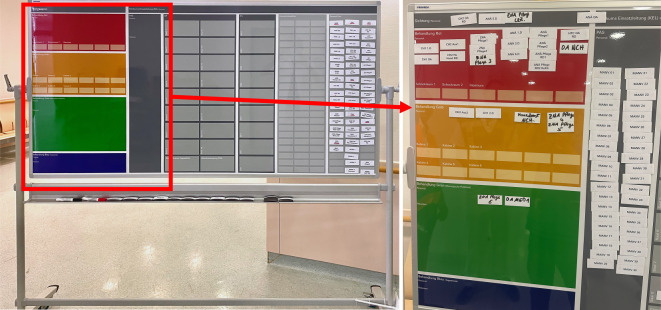
Spezielle, maßgefertigte Magnettafel zur Planung und zur Visualisierung des Einsatzgeschehens. Die Magnettafel bildet farbcodiert die verschiedenen Behandlungsbereiche und weiterbehandelnde Einheiten ab. Anhand von Magnetschildern erfolgt eine nachvollziehbare Zuordnung sowohl der Patienten als auch des Personals auf die Behandlungsbereiche. Die Magnettafel bietet so jederzeit eine Übersicht über die eingesetzten und noch frei vorhandenen personellen Ressourcen sowie den Patientenfluss

Zur Planung und zur Visualisierung des Einsatzgeschehens nutzte die opEL eine spezielle maßgefertigte Magnettafel, welche die verschiedenen Behandlungsbereiche (farbcodiert, Abb. 3) sowie weiterbehandelnde Einheiten wie OP und Intensivstationen abbildet, die anhand von entsprechenden Magnetschildern eine nachvollziehbare Zuordnung und Verteilung von Patienten und Personal auf die Behandlungsbereiche ermöglicht und eine Übersicht über die vorhandenen personellen Ressourcen sowie den Patientenfluss liefert [17, 19].

Den Behandlungsteams standen Patientenversorgungskarten (Zusatzmaterial online: ESM 1), auf welchen alle im Rahmen der Übung erhobenen Befunde und die theoretisch ergriffenen Maßnahmen vermerkt wurden, zur Verfügung. Zudem wurde jedem Patienten eine Versorgungsbox mit u. a. Infusionsbesteck, Verbandmaterial und Tourniquet zur Erstversorgung zugeteilt (Abb. 2).

Die Kommunikation zwischen der opEL und den Abschnittsleitern erfolgte über die Klinik internen DECT-Telefone.

Die Übung wurde mit Einverständnis aller Beteiligten videodokumentiert.

## Ergebnisse

Die Gesamtdauer der Übung betrug einschließlich Briefing und Debriefing fünf Stunden. An der Übung nahmen insgesamt 42 Klinikmitarbeitende folgender Abteilungen teil: Anästhesiologie, Allgemeinchirurgie, Herz-Thorax-Chirurgie, Innere Medizin, Logistik, Neurochirurgie, Medizintechnik, Unfallchirurgie, Radiologie und ZNA. Insgesamt waren 30 Schauspielpatienten an der Übung beteiligt, davon wurden 13 Patienten im UKW aufgenommen und behandelt. 7 Patienten wurden in SK I/rot, 2 in SKII/gelb und 4 in SK III/grün gesichtet.

**Abb. 4:**
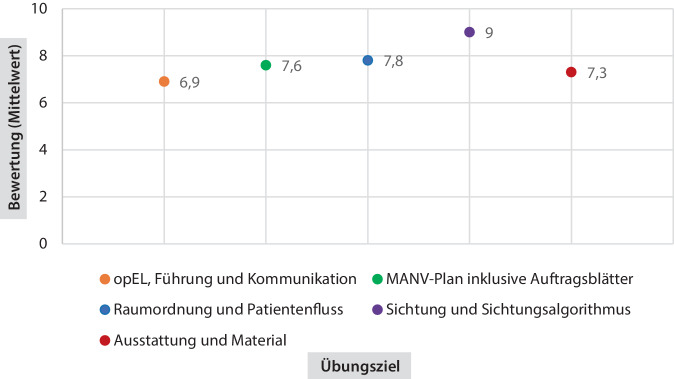
Erreichen der Übungsziele

**Abb. 5:**
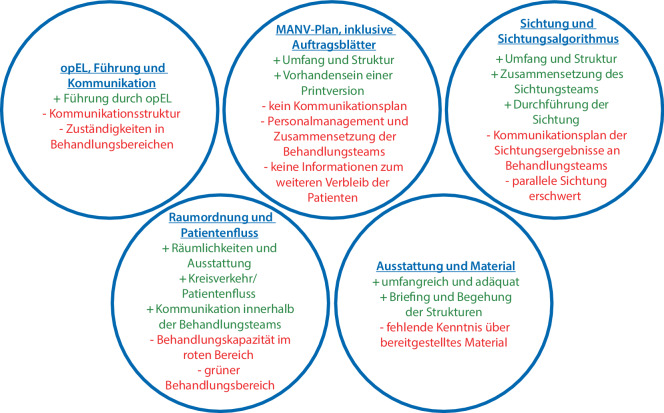
Auswertung der offenen Fragen

Das Erreichen der Übungsziele wurde anhand einer Likert-Skala bewertet (Abb. 4). Die Auswertung der offenen Fragen ist in Abb. 5 zusammengefasst.

### opEL, Führung und Kommunikation

Die opEL kommunizierte insgesamt gut mit den Sichtungs- und Behandlungsteams und hat den Einsatz adäquat geleitet.

Ein aktuelles und realistisches Lagebild ist hierbei essenziell für die Alarmierungsentscheidung und Einsatzplanung durch die opEL und KEL. Ein tatsächliches, umfangreiches Lagebild wurde erst spät im Verlauf der Übung seitens der Leitstelle an die opEL kommuniziert, sodass längere Zeit nicht klar war, mit welcher Anzahl an Patienten und welchen Verletzungsmustern zu rechnen ist.

Die Auswertung der Evaluationsbogen zeigte deutliche Defizite in der Kommunikationsstruktur, als wichtiges Schlüsselelement, auf. Die Kommunikation zwischen opEL und den Behandlungsteams sowie innerhalb der Behandlungsteams und -bereiche war durch folgende Punkte erschwert:

- keine klare Kommunikationsstruktur etabliert und kein Kommunikationsplan vorhanden (Wer spricht wann mit wem über was?),
- Unklarheiten über Zuständigkeiten innerhalb der Behandlungsteams,
- fehlende Kennzeichnung der Abschnittsleiter.

Damit sich der opEL-Leiter besser auf die Kommunikation und Einsatzleitung konzentrieren kann, wäre der Einsatz eines Führungsassistenten hilfreich gewesen. Dessen Aufgabe wäre die Führung des MANV-Boards gewesen.

### MANV-Plan, inklusive Auftragsblätter

Der MANV-Plan des Universitätsklinikums erwies sich als vollständig und gut geeignet, entsprechende Lagen zu strukturieren. Es zeigte sich jedoch noch an einigen Stellen Optimierungsbedarf. Beispielsweise fehlten im MANV-Plan:

- eine detaillierte Verfahrensanweisung zum Personalmanagement sowie zur Zusammensetzung und Funktion der Behandlungsteams,
- ein detaillierter Kommunikationsplan (s. oben).

Besonders positiv hervorgehoben wurde, dass die Auftragsblätter (AFB) als Printversion im MANV-Wagen vorgehalten werden. Dies sorgte für Handlungssicherheit auf den einzelnen Positionen. Auch haben die im Vorfeld der Übung organisierte Schulung und Einführung in die Raumordnung sowie vorgehaltenen Ressourcen zum Gelingen der Übung beigetragen und wurden von den Teilnehmenden als positiv bewertet. Insgesamt fehlten den Mitarbeitern jedoch die Übungspraxis und die allgemeine Kenntnis über das Vorhandensein und den Inhalt des MANV-Plans, der AFB und der vorgehaltenen Ressourcen.

### Raumordnung und Patientenfluss

Die Raumordnung im UKW ist klar strukturiert, und die entsprechenden Räumlichkeiten sind gut für die Patientenversorgung in den Bereichen rot und gelb ausgestattet. Die Behandlungskapazitäten im roten Bereich (3 Schockräume) sind jedoch bei hohem Patientenaufkommen schnell ausgelastet. Der grüne Bereich stellte sich als zu weit abgelegen und auch materiell eher unzureichend ausgestattet heraus. Eine Verlegung dieses Behandlungsbereichs in die materialtechnisch besser ausgestattete und näher gelegene internistische Spange der ZNA wurde als Verbesserungsmöglichkeit identifiziert.

Die Regelung des Kreisverkehrs ist aufwendig, garantiert jedoch einen geregelten Patientenfluss und verhindert ein Blockieren der Zu- und Abfahrt der ZNA.

Die Kommunikation innerhalb der Behandlungsteams war gut. Es herrschten jedoch Unklarheiten über die Personalbindung und den Abtransport der Patienten zu den nachbehandelnden Einheiten, und wie dieser von statten gehen soll.

Im Realfall wäre bei hohem Patientenaufkommen im Dienstgeschehen mit Personalengpässen zu rechnen, sodass frühzeitig an die Nachalarmierung freier Personalressourcen gedacht werden muss.

### Sichtung und Sichtungsalgorithmus

Der klinikinterne Sichtungsalgorithmus war gut geeignet zur Anwendung im Falle eines MANV. Das Sichtungsteam, bestehend aus erfahrenem ärztlichem und pflegerischem Personal, hat effizient, schnell und sicher gearbeitet. Eine zweite Pflege- oder Dokumentationskraft im Sichtungsteam wäre jedoch hilfreich gewesen. Aufgrund der engen Platzverhältnisse im Vorraum der ZNA ist die parallele Sichtung mehrerer Patienten erschwert. Da die Güte des klinikinternen Sichtungsalgorithmus nicht Gegenstand der Evaluation war, können keine Angaben zu dessen diagnostischer Qualität getätigt werden.

Die Kommunikation der Sichtungsergebnisse an die Behandlungsteams zeigte Defizite. So war den Behandlungsteams nicht immer ersichtlich, warum eine Einstufung in die jeweilige Sichtungskategorie erfolgte, bzw. welches Verletzungsmuster diese bedingte, da beispielsweise entsprechende Vermerke auf den Sichtungskarten fehlten.

### Ausstattung und Material

Das zur Verfügung stehende Material stellt sich zur Bewältigung von MANV-Lagen als gut geeignet dar, allerdings fehlt es teilweise an Kenntnis darüber, welche Materialien vorgehalten und wo diese gelagert werden. Eine im Vorfeld der Übung durchgeführte Begehung der Räumlichkeiten samt Ressourcen hat geholfen, dieses Problem anzugehen und die Vorkenntnisse des Personals über die zur Verfügung stehenden Mittel zu erhöhen.

## Diskussion

Die KAEP und der darin enthaltene MANV-Plan des Universitätsklinikum Würzburg sind grundsätzlich gut geeignet, einen MANV zu bewältigen, insbesondere wenn vorab geschult und trainiert wurde. Als zentrale Elemente der Evaluation konnten das Personalmanagement, die Kommunikation i. Allg. sowie der Schulungs- und Übungsbedarf der vorhandenen Pläne identifiziert werden.

Ein aktuelles und realistisches Lagebild ist essenziell für die Einsatzführung durch opEL und KEL. Um ein Unterangebot an Behandlungsplätzen im roten Bereich zu vermeiden, sollten die Prozesse hier möglichst schnell und effizient umgesetzt werden, frühzeitig die Kapazitäten in nachgeordneten Behandlungseinheiten wie OP-Bereich, Intensivstationen und Aufwachräume abgefragt und der Abtransport dorthin organisiert werden. Innerklinische Dienstressourcen sollten maximal ausgeschöpft werden und frühzeitig gezielt eine niederschwellige Alarmierung von weiterem Personal erfolgen, um Personalengpässe zu antizipieren.

Das Vorhandensein einer klaren Führungs- und Kommunikationsstruktur stellt in Großschadenslagen ein kritisches und erfolgsentscheidendes Element dar [3, 17]. Diese ist am UKW etabliert und bewährte sich im Rahmen der Übung. Die Kommunikation stellte sich auf mehreren Ebenen als verbesserungswürdig heraus. Zum einen fehlte es an einer im Vorfeld definierten Kommunikationsstruktur, zum anderen gab es Unklarheiten bezüglich der Zuständigkeiten in den Behandlungsbereichen. Um den Informationsfluss besser zu koordinieren, sollten im Vorfeld ein Kommunikationsplan erstellt und entsprechende Informationen zu Kommunikations- und Führungsstruktur in den MANV-Plan und die AFB eingepflegt werden. Laufkartensysteme eigenen sich zur Strukturierung und Optimierung der innerklinischen Abläufe im MANV-Fall [10, 16]. Zur Optimierung des Personalmanagements könnte daher, ergänzend zu den AFB, ein Laufkartensystem etabliert werden, wobei für jede Funktion spezifische Karten vorgehalten werden, welche Informationen zu individueller Funktion und Qualifikation der jeweiligen Person sowie zum Einsatzort, zu den Aufgaben sowie der Kommunikationshierarchie enthalten [10, 16]. Alle Funktionsträger (nicht nur KEL und Sichtung) sollten mittels Westen gekennzeichnet werden und mit eigens für den MANV vorgesehenen Kommunikationsmitteln ausgestattet werden. Die entsprechenden Rufnummern sollten im Kommunikationsplan sowie auf den AFB vermerkt werden. Die Kommunikation sollte als „closed-loop communication“ ablaufen. Der Kommunikationsplan sollte allen beteiligten Einsatzkräften als Schaubild zur Verfügung stehen.

Das Sichtungsteam muss aus erfahrenen Ärzten und Pflegekräften bestehen. Zudem sollte eine Dokumentationskraft das Sichtungsteam ergänzen [1, 13]. Der Sichtungsbogen sollte beim Patienten verbleiben und relevante, im Rahmen der Sichtung erlangte Erkenntnisse dort vermerkt werden, um einen Informationsverlust zu vermeiden und der erschwerten Kommunikation zwischen Sichtung und Behandlungsteams entgegenzukommen.

Es ist zielführend, sowohl im MANV-Plan als auch in AFB die Zusammensetzung der Behandlungsteams in den jeweiligen Bereichen sowie bezüglich der Personal- und Patientenbindung klar zu regeln. Vor allem in den kritischen Bereichen erscheint die Zuteilung des Personals in feste Teams (beispielsweise ein Anästhesist + ein Chirurg + Pflegekräfte) am Patienten, welche ihren Patienten nach der Sichtung empfangen, behandeln und bis zur weiterverhandelnden Einheit begleiten, sinnvoll. Dies wäre zeitgleich ein Lösungsansatz für die Probleme Übergabe, Informationsverlust und fehlende Rückkopplung über den Patientenverbleib [6, 7].

Gute Kenntnisse über die vorgehaltenen Materialien, die Inhalte der KAEP und eine konsequente Anwendung der im MANV-Plan und in den AFB festgelegten Prozesse sind essenziell für einen koordinierten Ablauf des Einsatzgeschehens. Die wenigsten Mitarbeiter sind jedoch ausreichend hierüber informiert und geschult. Um dieser Tatsache zu begegnen, ist es essenziell, regelmäßige Schulungen und Übungen zu implementieren, damit die Mitarbeiter die in der KAEP festgelegten Prozesse verinnerlichen und mit dem vorgehaltenen Material trainieren können [1, 2]. Zudem sollten sowohl MANV-Plan als auch AFB unkompliziert allen Mitarbeitern zum Nachschlagen zur Verfügung stehen (beispielsweise als Offline-App). Dass Übungen, Training und Schulung in einer verstetigten und fest verankerten Weise, v. a. an großen Krankenhäusern, eine extrem hohe Herausforderung darstellen und einen hohen Ressourcenaufwand fordern, darf kein Hinderungsgrund sein, erschwert die Etablierung aber ungemein.

## Fazit für die Praxis

- Ein Massenanfall von Verletzten stellt eine große Herausforderung für Krankenhäuser dar.
- Krankenhäuser müssen Alarm- und Einsatzpläne vorhalten, welche bei einem MANV aktiviert werden und einer akuten Überlastung begegnen sollen.
- Die in der KAEP festgehaltenen Abläufe und Pläne sollten regelmäßig trainiert und evaluiert werden. Das hier vorgestellte Konzept ist gut geeignet, die KAEP samt MANV-Plan lokal zu evaluieren und optimierungsbedürftige Elemente zu identifizieren.
- Klar definierte Zuständigkeiten und Verantwortlichkeiten, die Etablierung eines strukturierten Kommunikationsplans und die Erkennbarkeit von Funktionsträgern sind essenziell für die Bewältigung von MANV-Lagen.
- Die in der KAEP etablierten Prozesse sollten unter möglichst realen Bedingungen trainiert werden.

## Supplementary Information

ESM 1_Patientenversorgungskarte

ESM 2_Evaluationsbogen

## Abkürzungen

AFB: Auftragsblatt
DECT: Digital Enhanced Cordless Telecommunication
FAST: Focused Assessment with Sonography for Trauma
ITS: Intensivstation
KAEP: Krankenhausalarm- und -einsatzplanung
KEL: Krankenhauseinsatzleitung
MANV: Massenanfall von Verletzten
OpEL: Operative Einsatzleitung
SK: Sichtungskategorie
UKW: Universitätsklinikum Würzburg
ZNA: Zentrale Notaufnahme
ZOM: Zentrum Operative Medizin
