# Supplementary figures and images for: Evaluation of the hospital emergency plan based on an exercise for a mass casualty incident
Source: Anaesthesiologie. 2024 Oct 21;73(12):810–8. [Article in German] doi: 10.1007/s00101-024-01475-5 (PMC11615049; doi:10.1007/s00101-024-01475-5)

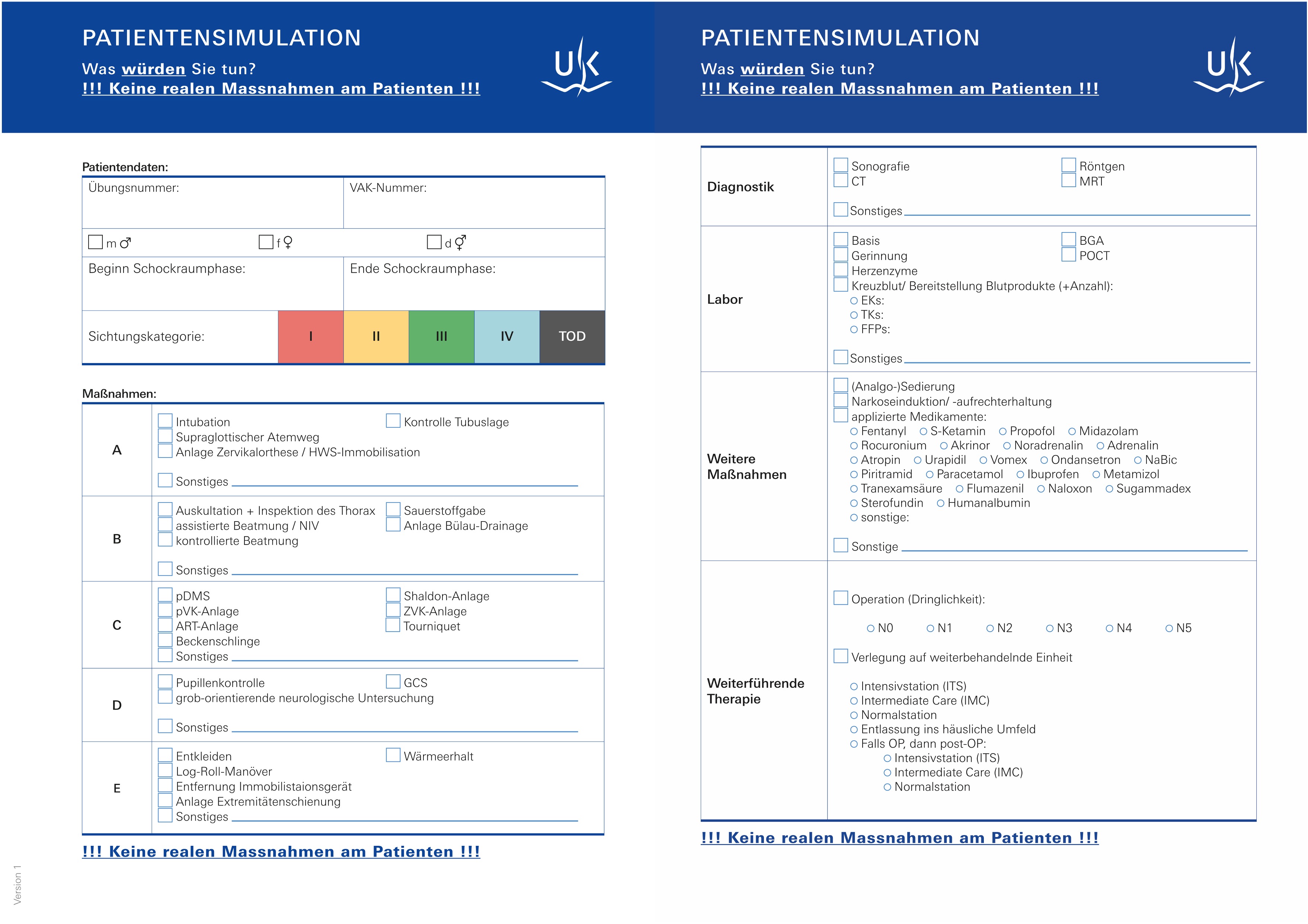

Supplement: Supplementary file 1 — ESM 1_Patientenversorgungskarte [file 101_2024_1475_MOESM1_ESM.jpg]

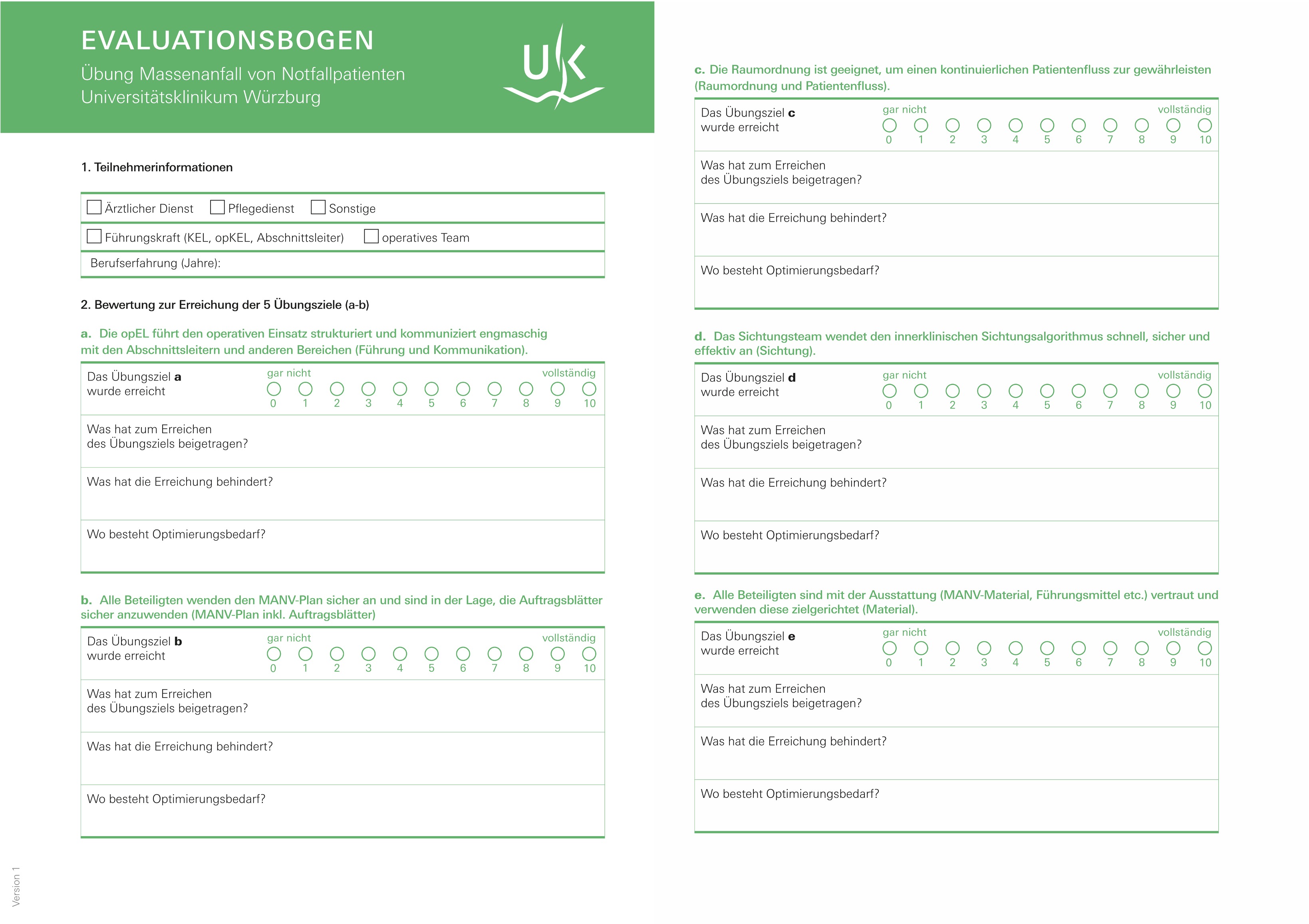

Supplement: Supplementary file 2 — ESM 2_Evaluationsbogen [file 101_2024_1475_MOESM2_ESM.jpg]
